# Molecular Epidemiology, Antifungal Susceptibility, and Virulence Evaluation of *Candida* Isolates Causing Invasive Infection in a Tertiary Care Teaching Hospital

**DOI:** 10.3389/fcimb.2021.721439

**Published:** 2021-09-15

**Authors:** Junzhu Chen, Niya Hu, Hongzhi Xu, Qiong Liu, Xiaomin Yu, Yuping Zhang, Yongcheng Huang, Junjun Tan, Xiaotian Huang, Lingbing Zeng

**Affiliations:** ^1^Department of Clinical Laboratory, The First Affiliated Hospital of Nanchang University, Nanchang, China; ^2^Department of Medical Microbiology, School of Medicine, Nanchang University, Nanchang, China; ^3^Department of Preventive Medicine and Public Health, School of Public Health, Nanchang University, Nanchang, China

**Keywords:** *Candida* species, RAPD, caseinase, hemolysin, biofilm, antifungal susceptibility

## Abstract

**Background:**

The incidence of invasive candidiasis is increasing worldwide. However, the epidemiology, antifungal susceptibility, and virulence of *Candida* spp. in most hospitals remain unclear. This study aimed to evaluate invasive candidiasis in a tertiary care hospital in Nanchang City, China.

**Methods:**

MALDI-TOF MS and 18S rDNA ITS sequencing were used to identify *Candida* strains. Randomly amplified polymorphic DNA analysis was used for molecular typing; biofilm production, caseinase, and hemolysin activities were used to evaluate virulence. The Sensititre™ YeastOne YO10 panel was used to examine antifungal susceptibility. Mutations in *ERG11* and the hotspot regions of *FKS1* of drug-resistant strains were sequenced to evaluate the possible mechanisms of antifungal resistance.

**Results:**

We obtained 110 *Candida* strains, which included 40 *Candida albicans* (36.36%), 37 C. *parapsilosis* (33.64%), 21 C. *tropicalis* (19.09%), 9 C. *glabrata* (8.18%), 2 C. *rugose* (1.82%), and 1 C. *haemulonii* (0.91%) isolates. At a limiting point of 0.80, *C*. *albicans* isolates could be grouped into five clusters, *C*. *parapsilosis* and *C*. *tropicalis* isolates into seven clusters, and *C*. *glabrata* isolates into only one cluster comprising six strains by RAPD typing. Antifungal susceptibility testing revealed that the isolates showed the greatest overall resistance against fluconazole (6.36%), followed by voriconazole (4.55%). All *C*. *albicans* and *C*. *parapsilosis* isolates exhibited 100% susceptibility to echinocandins (i.e., anidulafungin, caspofungin, and micafungin), whereas one *C*. *glabrata* strain was resistant to echinocandins. The most common amino acid substitutions noted in our study was 132aa (Y132H, Y132F) in the azole-resistant strains. No missense mutation was identified in the hotpot regions of *FKS1*. Comparison of the selected virulence factors detectable in a laboratory environment, such as biofilm, caseinase, and hemolysin production, revealed that most *Candida* isolates were caseinase and hemolysin producers with a strong activity (Pz < 0.69). Furthermore, *C. parapsilosis* had greater total biofilm biomass (average Abs_620_ = 0.712) than *C. albicans* (average Abs_620_ = 0.214, p < 0.01) or *C. tropicalis* (average Abs_620_ = 0.450, *p* < 0.05), although all *C. glabrata* strains were either low- or no-biofilm producers. The virulence level of the isolates from different specimen sources or clusters showed no obvious correlation. Interesting, 75% of the *C. albicans* from cluster F demonstrated azole resistance, whereas two azole-resistant *C. tropicalis* strains belonged to the cluster Y.

**Conclusion:**

This study provides vital information regarding the epidemiology, pathogenicity, and antifungal susceptibility of *Candida* spp. in patients admitted to Nanchang City Hospital.

## Introduction

Fungal infections in humans are relatively common and range from common or mild superficial infections to life-threatening invasive infections. In recent years, the widespread use of broad-spectrum antibiotics, hormones, immunosuppressive agents, chemotherapy, and central venous catheters have led to an increase in the incidence of invasive fungal infections. More than 1.6 million people worldwide have been reported to suffer from serious fungal diseases that have a profound impact on patients and can even be fatal (Bongomin et al., 2017).

Infections caused by *Candida* spp. can be divided into superficial, cutaneous, mucosal, and invasive infections (deep and extensive). Up to date, *Candida* spp. have become the third most common cause of bloodstream infections, including candidemia (Wisplinghoff et al., 2004). *Candida albicans* is responsible for approximately 50% of all candidiasis, and the other species account for the remaining *Candida* infections. Specifically, infections caused by *C*. *tropicalis*, *C*. *glabrata*, *C*. *parapsilosis*, *C*. *krusei*, and *C*. *auris* have attracted significant scientific attention (Mba and Nweze, 2020). Invasive candidiasis has been attributed to 40%–50% mortality in intensive care units (ICUs) (De Rosa et al., 2009). Candidemia is currently the fourth most common nosocomial bloodstream infection in America (Wisplinghoff et al., 2004; Li et al., 2016). In Europe, the incidence of candidemia was estimated to be approximately 79 cases per day, of which 29 patients were estimated to have a fatal outcome on Day 30 (Koehler et al., 2019).

As the mortality rate in candidiasis is high, identifying the relationship among the various *Candida* species and assessing disease epidemiology, species distribution, and drug resistance to facilitate the control of nosocomial candidiasis is crucial. This study describes the results from an analysis of 110 *Candida* strains isolated from sterile body fluid samples obtained between 2014 and 2019 from patients in a tertiary hospital in Nanchang City. *Candida* spp. in the samples were identified, tested, and analyzed to evaluate the prevalence of *Candida* infection and determine virulence profiles.

## Materials and Methods

### Strains

This retrospective study was conducted between September 2014 and September 2019. *Candida* spp. were isolated from sterile fluid samples (i.e., blood, ascites, bile, secretions, and abscess) of patients admitted in the First Affiliated Hospital of Nanchang University, China. The ethics committee of the university hospital approved this study (approval no. 2014036).

### Separation and Identification

The desired strains were isolated and purified on Sabouraud’s dextrose agar (SDA; 1% peptone, 4% dextrose, and 2% agar) plates and cultured at 30°C for 24 h. The first crucial step in our study is the correct identification of the *Candida* species. The strains were initially identified by MALDI-TOF mass spectrometry analyses using a Clin-ToF-II mass spectrometer (Bioyong, China). To ensure accuracy, 18S rDNA ITS sequencing was performed for further identification. Briefly, the contiguous ITS1-5.8S rDNA-ITS2 region was amplified with the universal fungal primers ITS1 (5′-TCCGTAGGTGAACCTGCGG-3′) and ITS4 (5′-TCCTCCGCTTATTGATATGC-3′) (Salehi et al., 2017). PCR reactions were performed using the Bio-Rad S1000 thermal cycler in 50 μL volumes containing 1 μL of extracted DNA, 47 μL of T3 Super PCR mix (TSINGKE, China), and 1 μL of each ITS1 and ITS4 primers. Amplification was performed with cycles of 3 min at 98°C for primary denaturation, followed by 35 cycles at 98°C (10 s), 55°C (10 s), and 72°C (20 s), along with a final extension at 72°C for 5 min. The PCR amplification products were sequenced by Tsingke Biological Company (Changsha, China). The sequences were analyzed using the NCBI BLAST searches (http://blast.ncbi.nlm.nih.gov/Blast.cgi).

### Randomly Amplified Polymorphic DNA Analysis

DNA from individual strains was extracted as described by Jain et al. but with certain modifications (Jain et al., 2001). Briefly, overnight-cultured *Candida* strains were harvested *via* centrifugation at 12000 rpm after washing with sterile PBS, and the precipitate was resuspended in 100 μL STES buffer (0.2 M Tris-HCl, 0.5 M NaCl, 0.1% SDS, and 0.01 M EDTA, pH = 8.0) and 20 μL TE buffer (0.01 M Tris-HCl and 0.001 M EDTA, pH = 8.0). Acid-washed glass beads (Sigma-Aldrich, USA) and 120 μL phenol–chloroform–isoamyl alcohol mixture (25:24:1 v/v/v) were added to lyse cells and release DNA. After 10 min of vortexing, lysates were centrifuged at 12000 rpm and 4°C for 10 min. DNA from the aqueous phase was precipitated with 2 times the volume of absolute ethanol at −20°C for 15 min, centrifuged at 12000 rpm and 4°C for 10 min, dried, and resuspended in 50 μL TE buffer. The concentration of nucleic acids was measured using a NanoDrop-2000 spectrophotometer (Thermo Fisher Scientific, USA).

Genomic DNA was amplified using previously described primers for five genes, namely, CD16AS (5′-CTCTTGAAACTGGGGAGACTTGA-3′), HP1247 (5′-AAGAGCCCGT-3′), ERIC-2 (5′-AAGTAAGTGACTGGGGTGAGCG-3′), OPE-3 (5′-CCAGATGCAC-3′), and OPE-18 (5′-GGACTGCAGA-3′) (Paluchowska et al., 2014). The primers used were synthesized by the Tsingke Biological Technology Company. Randomly amplified polymorphic DNA (RAPD) reactions were performed in a final volume of 50 μL, which included 1 μL of the DNA template (approximately 50 ng), 1 μL of each primer (10 μM), and the T3 Super PCR mix. Amplification reactions were performed for 40 cycles using a Bio-Rad S1000 thermal cycler under the following conditions: initial denaturation at 98°C for 3 min, followed by denaturation at 98°C for 10 s, annealing at 36°C for 1 min, and extension at 72°C for 2 min, along with a final extension at 72°C for 5 min. The DNA fragments were separated by electrophoresis in a 1% agarose gel run at 100 V for 30 min. Fragment sizes were determined by comparison with a DL5000 DNA Marker (Vazyme, China), and bands were visualized in the ChemiDocXRS+ system (Bio-Rad, USA). Similarity analysis of RAPD patterns was performed using NTSYS 2.10 software.

### Drug Sensitivity Test

*In vitro* susceptibility of *Candida* strains to nine antifungal drugs, namely, 5-flucytosine, anidulafungin, caspofungin, itraconazole, micafungin, posaconazole, voriconazole, amphotericin B, and fluconazole, was determined using the Sensititre™ YeastOne YO10 panel (Thermo Scientific, USA) based on manufacturer’s instructions. *C*. *krusei* ATCC 6258 and *C*. *parapsilosis* ATCC 22019 were used as controls, as per the Clinical and Laboratory Standards Institute guidelines. Plates were incubated at 35°C for 24 h, and minimum inhibitory concentrations (MICs) were interpreted according to documents M27-A3 and M27-S4 (Institut, C.a.L.S 2008; Institute, C.a.L.S 2012).

### Sequencing of *ERG11* and *FKS1*

The entire open reading frame (ORF) of *ERG11* from azole-resistant strains were amplified and sequenced with specific primers (**Supplementary Table S1**). The hotspot 1 (HS1) and hotspot 2 (HS2) sequences of *FKS1* were amplified using PCR with specific primers shown in **Supplementary Table S1** based on their key role in echinocandin resistance. Conventional PCRs were performed in a 50 μL reaction mixture containing 47 μL of T3 Super PCR mix, 0.2 μM of each primer, and 100 ng of the extracted DNA. The amplification conditions were as follows: initial denaturation at 98°C for 3 min followed by 35 cycles of denaturation (98°C, 10 s), annealing (52°C, 20 s), and extension (72°C, 5 min), followed by a final extension of 72°C for 10 min. The PCR products were purified and sequenced by the Tsingke Biological Technology Company. The *ERG11* sequences were aligned using BLAST and compared with the related published GenBank sequence for *C. albicans* SC5314 (Gene ID: 3641571), *C. parapsilosis* ATCC 22019 (Gene ID GQ302972.1), *C. tropicalis* ATCC 750 (Gene ID: M23673), *C. glabrata* CBS138 (Gene ID: XM 445876), and *C. haemulonis* (Gene ID: XM_025486744.1). The *FKS1* sequences were compared with the reference sequences of *C. tropicalis* ATCC 750 (Gene ID: EU676168) and *C. glabrata* (Gene ID: XM_446406).

### Caseinase Activity

Caseinase activity was measured by the single diffusion technique in SDA plates containing 1% casein (Ramesh et al., 2011). A standard inoculum (10^6^ cells/mL) was prepared in saline solution from an overnight yeast culture for each isolate and 10 μL of this standard inoculum was plated. Plates were incubated at 37°C for 5 days. Three independent replicates were tested for each strain. Colony diameter (a) and colony diameter plus precipitation zone (b) were measured using a vernier caliper (Guanglu, China). Phospholipase index (defined as Pz = a/b, based on the study by Price et al) was used to evaluate the extent of hydrolytic enzyme production by various *Candida* species (Price et al., 1982). According to this definition, a lower Pz value indicates a greater enzymatic activity, which was scored into four categories as follows: Pz = 1.00 was defined as no enzymatic activity, Pz = 0.90–0.99 as weak enzymatic activity, Pz = 0.89–0.70 as moderate activity, and Pz < 0.69 as strong enzymatic activity (Price et al., 1982).

### Hemolysin Activity

Hemolysin assay for *Candida* strains was measured using blood agar plates that were prepared by adding 6% human blood to 100 mL of SDA supplemented with 3% glucose (final concentration, wt/vol; pH = 5.6) (Neji et al., 2017). Standard inoculum (10 μL of 10^6^ cells/mL) was spotted onto the blood plates, plates were incubated at 37°C for 5 days, and hemolytic activity was measured and evaluated using the method described by Price et al. (1982). The assay was performed in triplicate for each isolate.

### Biofilm Formation

Biofilm production in *Candida* species was evaluated using a modified crystal violet assay described elsewhere (Silva et al., 2009; Mancera et al., 2015; Neji et al., 2017). Briefly, biofilm formation was spectrophotometrically determined wherein each well of a 96-well polystyrene plate was treated with 200 μL of 5% BSA (dissolved in sterilized PBS) at 4°C for 48 h and washed once with sterile PBS. Each experimental condition was designed such that five replicates were distributed in one column of the microplate. Sterility controls, i.e., without cell suspension, were used as a negative control to ensure no accidental contamination during treatment. Clinical isolates and the control strain Sc5314 were first cultured on SDA plates at 30°C for 24 h and then in 5 mL SDA medium overnight at 30°C with shaking. Cells were collected by centrifugation at 2458 g for 5 min, washed twice with sterilized PBS, and resuspended in spider medium (20 g/L nutrient broth, 20 g/L mannitol, and 4 g/L K_2_HPO4; pH 7.2). The turbidity of each strain suspension was spectrophotometrically adjusted to OD_600_ = 0.5; 200 μL of the cell suspension (OD_600_ = 0.5) was transferred to each well of the 96-well plates, which were incubated at 37°C for 90 min. Next, the strain suspension was removed, 200 μL of fresh spider medium was added, and the plates were incubated at 37°C for 48 h without shaking (static culture). Plates were washed twice with PBS to remove nonadherent cells after the adhesion stage and biofilms were fixed by incubation with 200 μL methanol for 30 min. Afterward, the plates were dried at room temperature and 200-μL 1% crystal violet (Solarbio, China) was added to each well and incubated for 30 min. The wells were then gently washed with water until the washing was colorless and incubated with 200 µL of acetic acid for 1 h to dissolve the biofilm. The absorbance of the obtained solution was read at 620 nm in a microtiter plate reader (Molecular Devices, USA). Absorbance values of the controls were subtracted from test values to eliminate background interference.

### Statistical Analysis

Data were analyzed with SPSS (version 25.0) and GraphPad Prism (version 5.0). Unless otherwise mentioned, data are presented as mean ± standard deviation (SD). The normality of data distribution was evaluated using the D’ Agostino-Pearson omnibus normality test. Differences between two independent groups were performed with the nonparametric Mann–Whitney U test or the Kolmogorov–Smirnov Z test for the relative values. A *p* value of ≤0.05 was defined as statistically significant.

## Results

### Prevalence of Invasive *Candida* spp. Isolates

A total of 105 patients were enrolled in this study. The study population comprised 77 (73.3%) males and 28 (26.7%) females with a median age of 53 years (range 0–87 years). Although most isolates were obtained from patients hospitalized in the ICU (n = 31, 28.18%), 26 (23.6%) came from the department of gastroenterology, 14 (12.73%) from the burns unit, 11 (10.0%) from emergency services unit, 5 (4.54%) from infectious diseases unit, 4 (3.6%) from the hematology department, and 17 (15.5%) from other non-ICU departments (**Table 1**). Overall, 22.9% (24/105) of the patients died, mainly from acute pancreatitis, which accounted for approximately 37.5% of all mortality cases. Antifungal use (oral or injected) was reported for 42.9% (45/105) of the patients. In our study, the most common antifungal drug used was fluconazole (44.4%, 20/45), followed by voriconazole (40.0%, 18/45) and caspofungin (15.6%, 7/45).

**Table 1:** Distribution of *Candida* isolates by a source of isolation.

	***C. albicans***	***C. parapsilosis***	***C. tropicalis***	***C. glabrata***	***C. rugosa***	***C. haemulonii***	**Total**
ICUs	9 (8.18%)	11 (10.00%)	4 (3.64%)	6 (5.45%)	1 (0.91%)	0	31 (28.18%)
Department of gastroenterology	12 (10.91%)	7 (6.36%)	5 (4.54%)	2 (1.82%)	0	0	26 (23.64)
Department of burn wound	5 (4.54%)	4 (3.64%)	4 (3.64%)	0	1 (0.91%)	0	14 (12.73)
Department of emergency	6 (5.45%)	1 (0.91%)	3 (2.73%)	1 (0.91%)	0	0	11 (10.00%)
Department of infectious diseases	0	4 (3.64%)	1 (0.91%)	0	0	0	5 (4.54%)
Department of hematology	0	0	4 (3.64%)	0	0	0	4 (3.64%)
Department of orthopedics	1 (0.91%)	1 (0.91%)	0	0	0	0	2 (1.82%)
Department of urology	3 (2.73%)	0	0	0	0	0	3 (2.73%)
Department of general surgery	1 (0.91%)	2 (1.82%)	0	0	0	0	3 (2.73%)
Department of Nephrology	0	2 (1.82%)	0	0	0	0	2 (1.82%)
Department of neonatal pediatrics	1 (0.91%)	1 (0.91%)	0	0	0	1 (0.91%)	3 (2.73%)
Department of ENT	1 (0.91%)	0	0	0	0	0	1 (0.91%)
Department of maternity	1 (0.91%)	0	0	0	0	0	1 (0.91%)
Plastic Surgery	0	1 (0.91%)	0	0	0	0	1 (0.91%)
Cardiothoracic Surgery	0	3 (2.73%)	0	0	0	0	3 (2.73%)
Total	40 (36.36%)	37 (33.64%)	21 (19.09%)	9 (8.18%)	2 (1.82%)	1 (0.91%)	110 (100%)

**Figure 1:**
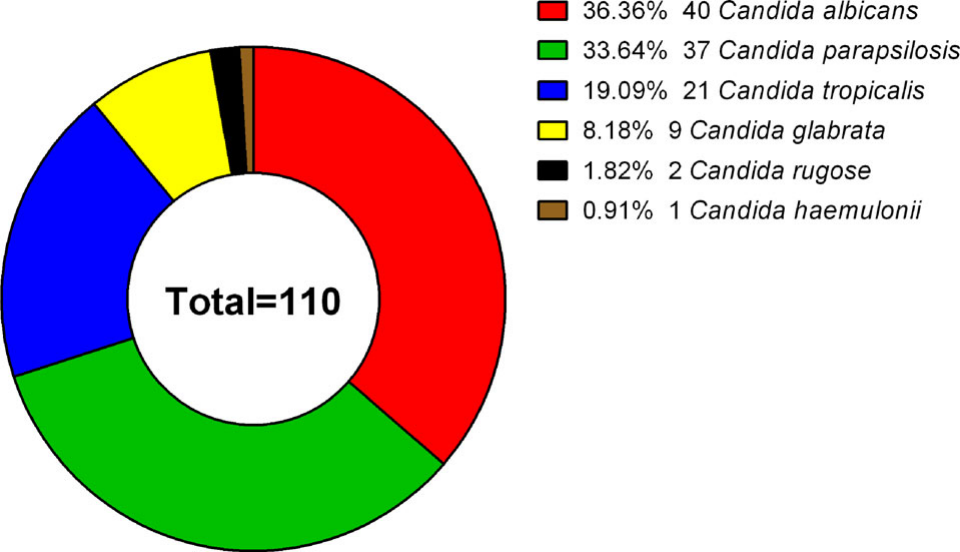
Species distribution of 110 clinical isolates. The 110 *Candida* strains analyzed included 40 C*. albicans* (36.36%), 37 C*. parapsilosis* (33.64%), 21 C*. tropicalis* (19.09%), 9 C*. glabrata* (8.18%), 2 C. rugose (1.82%), and 1 C*. haemulonii* (0.91%) isolate.

A total of 110 clinical isolates (40 C*. albicans*, 37 C*. parapsilosis*, 21 C*. tropicalis*, 9 C. *glabrata*, 2 C. *rugose*, and 1 C. *haemulonii* isolates) were studied (**Figure 1**). Each isolate was obtained from different patients, with the following exceptions: *C. albicans* NCU_B040, NCU_B049, and NCU_B059, and *C. tropicalis* NCU_B047 and NCU_B050 strains, were acquired from the blood of one patient and three strains of *C*. *glabrata* (NCU_B103, NCU_O109, and NCU_O145) were isolated from different source sites of the same patient.

**Figure 2:**
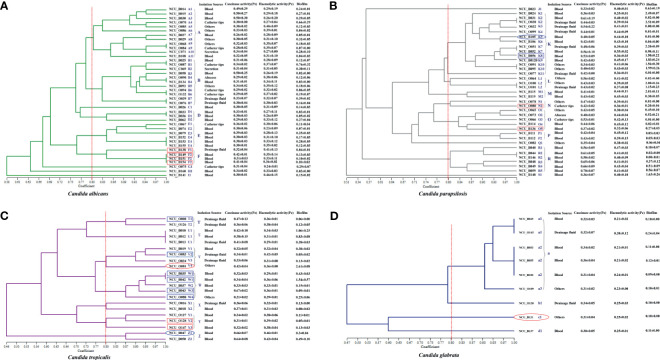
Dendrogram presenting the genetic relatedness of 107 *Candida* spp. based on the random amplified polymorphic DNA (RAPD) data. The virulence factors (i.e., caseinase, hemolysin, and biofilm), drug resistance, and specimen sources are indicated in the figure. The vertical line divides the strains based on the level of genetic similarity into related and unrelated strains. The red boxes indicate azole resistance (fluconazole and voriconazole), whereas the blue boxes indicate azole-susceptible (S) dose dependence (SDD). Red ellipses represent resistance to echinocandins, while the blue ellipses represent intermediate resistance to micafungin. **(A)** Dendrogram of 40 *C. albicans* isolates. **(B)** Dendrogram of 37 *C. parapsilosis* isolates. **(C)** Dendrogram of 21 *C. tropicalis* isolates. **(D)** Dendrogram of 9 *C. glabrata* isolates.

Sources of the 110 isolates (**Table 2**) were predominantly blood (n = 64, 58.18%), followed by catheter tips (n = 9, 8.18%) and drainage fluid (n = 17, 15.45%), and also included abscesses (1.82%), secretions (1.82%), and other sources (14.55%). Furthermore, as shown in **Table 2** and **Figure 2**, *C*. *albicans* could be isolated from a wider range of specimens than other species. However, *C. haemulonii* and *C. rugose* were isolated only from blood.

**Table 2:** Comparison of the virulence factors among *Candida* strains isolated from different sources (mean optical density ± SD).

		*C. albicans*			*C. parapsilosis*			*C. tropicalis*			*C. glabrata*			*C. rugosa*		*C. haemulonii*	
		N	Mean Abs ± SD	Range	N	Mean Abs ± SD	Range	N	Mean Abs ± SD	Range	N	Mean Abs ± SD	Range	N	Range	N	Range
Blood (N=64)	Biofilm	22	0.139 ± 0.094	0.027-0.295	21	0.646 ± 0.778	0.007-2.492	13	0.438 ± 0.454	0.059-1.546	5	0.112 ± 0.011	0.095-0.123	2	0.085-0.098	1	0.061-0.061
	Caseinase		0.357 ± 0.078	0.257-0.504		0.484 ± 0.112	0.330-0.703		0.417 ± 0.139	0.310-0.669		0.325 ± 0.024	0.300-0.355		0.516-0.657		0.453-0.453
	Hemolysin		0.309 ± 0.047	0.233-0.456		0.420 ± 0.135	0.325-0.555		0.357 ± 0.049	0.287-0.457		0.221 ± 0.019	0.207-0.246		0.514-0.555		0.336-0.336
Catheter tips (N=9)	Biofilm	7	0.312 ± 0.291	0.065-0.768	2	0.110 ± 0.141	0.010-0.209	0	0		0	0		0		0	0
	Caseinase		0.311 ± 0.031	0.277-0.363		0.473 ± 0.076	0.419-0.527										
	Hemolysin		0.318 ± 0.052	0.336-0.373		0.390 ± 0.042	0.360-0.420										
Drainage fluid (N=17)	Biofilm	3	0.203 ± 0.181	0.040-0.399	6	0.986 ± 1.157	0.004-2.514	6	0.150 ± 0.121	0.051-0.358	2	0.178 ± 0.098	0.109-0.247	0		0	
	Caseinase		0.316 ± 0.016	0.298-0.329		0.456 ± 0.045	0.415-0.536		0.380 ± 0.053	0.330-0.471		0.328 ± 0.015	0.317-0.338				
	Hemolysin		0.347 ± 0.059	0.304-0.414		0.394 ± 0.028	0.361-0.440		0.348 ± 0.049	0.287-0.418		0.313 ± 0.090	0.249-0.376				
Abscess (N=2)	Biofilm	1	0.127	0.127-0.127	1	0.522	0.522-0.522	0	0		0	0		0	0		
	Caseinase		0.295	0.295-0.295		0.403	0.403-0.403										
	Hemolysin		0.383	0.383-0.383		0.44	0.440-0.440										
Secretion (N=2)	Biofilm	2	0.296 ± 0.010	0.289-0.303	0	0	0	0	0		0	0		0	0		
	Caseinase		0.325 ± 0.017	0.313-0.337													
	Hemolysin		0.291 ± 0.024	0.274-0.308													
Others (N=16)	Biofilm	5	0.174 ± 0.103	0.049-0.328	7	0.876 ± 0.940	0.016-2.006	2	1.432 ± 1.667	0.253-2.611	2		0.109-0.109	0	0		
	Caseinase		0.319 ± 0.018	0.300-0.344		0.425 ± 0.072	0.345-0.558		0.365 ± 0.083	0.306-0.423		0.309 ± 0.004	0.306-0.311				
	Hemolysin		0.358 ± 0.034	0.312-0.396		0.389 ± 0.042	0.302-0.434		0.374 ± 0.024	0.357-0.391		0.241 ± 0.014	0.231-0.251				

N, number of tested isolates; SD, standard deviation.

### Molecular Phylogenetic Analysis of the Invasive *Candida* Spp. Isolates

To explore the diversity of the isolated *Candida* spp., RAPD analysis of the four major species of clinical isolates was performed with five random primers using the UPGMA method. *C. rugose* and *C. haemulonii* strains were not analyzed because of their low isolation rates. The number of patterns generated by *C. albicans*, *C. parapsilosis*, *C. tropicalis*, and *C. glabrata* isolates with each primer changed between 3–10, 4–12, 3–10, and 6–10, respectively. All primers were used for the dendrogram construction.

We recognized 32 distinct RAPD profiles among the 40 C. *albicans* isolates analyzed. **Figure 2A** shows the cluster analysis of the electrophoretic bands of the *C*. *albicans* isolates, and the Sj value ranged from 0.58 to 1.00. Four strains presented unique patterns (C1, G1, H1, and I1) and were considered to be unrelated. At a limiting point of 0.80, 36 C. *albicans* isolates could be allocated into five clusters (A, B, and D–F). For *C*. *parapsilosis*, the primers produced up to 12 bands, and therefore, this set displayed the greatest discriminatory power. In their dendrogram, *C*. *parapsilosis* isolates could be grouped into seven clusters (K–P, and R) when 0.80 was assumed as the limiting value, whereas three isolates (NCU_B023, NCU_O082, and NCU_B148) were deemed unrelated (**Figure 2B**). Among the 21 C. *tropicalis* isolates, 18 RAPD profiles could be distinguished, and they were accommodated into seven clusters (T–Z) (**Figure 2C**). Six genotypes (a1– a3, b1, c1, and d1) were observed upon the RAPD analysis of the nine *C*. *glabrata* isolates. A total of 6 C*. glabrata* isolates with a similarity level >80% were allocated into the same cluster; of these, NCU_B103, NCU_O109, and NCU_O145 were different sites of the same patient and were grouped in the same cluster. Three genotypes (b1, c1, and d1) were represented by only one isolate each, and they were considered unrelated (**Figure 2D**).

### Antifungal Susceptibility for the Invasive *Candida* spp. Isolates

**Table 3** shows the susceptibility profiles of the *Candida* species to the nine antifungal drugs tested. MICs were also determined for two rare yeast species (one *C*. *haemulonii* and two *C*. *rugose* isolates); however, fewer than five isolates were studied for these two *Candida* spp. Furthermore, drug-resistant strains of *C*. *haemulonii* and *C*. *rugose* could not be accurately determined because of a lack of validated clinical breakpoints. Nevertheless, on the basis of CLSI document M27-S4, we found that nine isolates were resistant to azoles. The MIC_90_ values for fluconazole were 1–2, 1, 4, and 32 μg/mL for *C*. *albicans*, *C*. *parapsilosis*, *C*. *tropicalis*, and *C*. *glabrata*, respectively. MIC_90_ values for voriconazole, itraconazole, and posaconazole were in the range of ≤0.08–1, 0.12–1, and 0.06–2 μg/mL, respectively, for *C*. *albicans*, *C*. *parapsilosis*, *C*. *tropicalis*, and *C*. *glabrata*, respectively. In addition, MIC for fluconazole ranged from 4 to 16 μg/mL for *C*. *rugose* isolates, whereas that for *C*. *haemulonii* was high at 128 μg/mL. MIC_90_ values of echinocandins (anidulafungin, caspofungin, and micafungin) against *C*. *parapsilosis* were higher than those for *C*. *albicans*, *C*. *tropicalis*, and *C*. *glabrata*.

**Table 3:** Minimum inhibitory concentration values (μg/mL) for the *Candida* isolates.

Antifungals	*C. albicans*			*C. parapsilosis*			*C. tropicalis*			*C. glabrata*			*C. rugosa*	*C. haemulonii*
	MIC range	MIC50	MIC90	MIC range	MIC50	MIC90	MIC range	MIC50	MIC90	MIC range	MIC50	MIC90	MIC range	MIC range
Anidulafungin	≤0.015-0.12	0.03	0.12	0.06-2	1	1	0.03-2	0.12	0.25	≤0.015-0.12	0.03	0.06	0.25-2	0.06-0.06
Caspofungin	0.015-0.25	0.06	0.12	0.03-2	0.5	1	0.06-0.5	0.06	0.12	0.03-0.5	0.06	0.06-0.12	0.5-8	0.06-0.06
Micafungin	≤0.008-0.12	≤0.008	0.015	0.12-4	1	1	0.015-2	0.03	0.06	≤0.008-2	0.015	0.015	0.06-0.5	0.12-0.12
Fluconazole	≤0.12-32	0.25	1-2	0.5-64	0.5	1	0.5->256	2	4	1-32	16	32	4-16	128-128
Voriconazole	≤0.008-1	≤0.008	≤0.008-1	≤0.008-2	≤0.008	0.03	≤0.008->8	0.12	0.25	0.06-2	1	1	0.06-0.25	8-8
Itraconazole	≤0.015-0.12	0.03-0.06	0.12	≤0.015-0.5	0.06-0.12	0.12	0.03-1	0.25	0.5	0.12-2	1	1	0.12-0.25	16-16
5-Flucytosine	≤0.06-8	≤0.06	0.12	≤0.06-0.5	≤0.06	≤0.06	≤0.06-0.12	≤0.06	≤0.06	≤0.06-0.5	≤0.06	≤0.06	0.12-0.25	64-64
Amphotericin B	≤0.12-0.5	0.5	0.5	≤0.12-2	0.25	0.25	≤0.12-1	0.5	1	≤0.12-2	0.5	0.5-1	0.5-1	0.5-0.5
Posaconazole	≤0.008-1	0.015-0.03	0.06	0.015-0.5	0.06	0.06	0.015-1	0.25	0.5	0.5-8	2	2	0.06-0.12	8-8

**Table 4** shows the overall drug susceptibility pattern of these four *Candia* species against the five antifungal drug types tested. All *C*. *albicans* and *C*. *parapsilosis* isolates exhibited 100% of susceptibility to echinocandins, whereas one strain of *C*. *glabrata* was resistant (**Table 4**). Overall resistance was greatest against fluconazole (6.36%), followed by voriconazole (4.55%). As far as species-specific antifungal resistance rates were concerned, *C*. *tropicalis* was resistant to almost all drugs tested (except for caspofungin), with the least resistance rate of 4.76%. Isolates of *C*. *tropicalis* were susceptible to fluconazole and voriconazole with sensitivity rates of 80.95% and 61.90%, respectively. It is worth noting that the current data were insufficient for distinguishing between drug-resistant and -sensitive strains of *C*. *glabrata* based on the CLSI document M27-S4 (Institute, 2012). One (11.11%) of the nine *C*. *glabrata* strains was resistant to echinocandins, and all *C*. *glabrata* isolates met the criteria for “susceptible-dose dependence (S-DD)” for fluconazole (**Table 4**).

**Table 4:** Antifungal susceptibility testing results of four main *Candida* species against five antifungal drugs.

Antifungal	*C. albicans* (N = 40)				*C. parapsilosis* (N = 37)				*C. tropicalis* (N = 21)				*C. glabrata* (N = 9)			
	S	S-DD	I	R	S	S-DD	I	R	S	S-DD	I	R	S	S-DD	I	R
Anidulafungin	40		0	0	37		0	0	20		0	1	8		0	1
Caspofungin	40		0	0	37		0	0	20		1	0	8		0	1
Micafungin	40		0	0	36		1	0	19		1	1	8		0	1
Fluconazole	37	0		3	34	1		2	17	2		2	0	9	0	0
Voriconazole	37	1		2	35	1		1	13	6		2				

S, susceptible; SDD, susceptible-dose dependent; I, intermediate; R, resistant.

### Mutations In *ERG11* and the HS Regions of *FKS1*

Comparison of the complete ORF of *ERG11* of the eight isolates with that of the published wild-type sequences revealed 12 mutations, of which five were silent without any amino acid changes (data not shown). Seven missense mutations were detected in these seven strains. Of the seven distinct amino acid substitutions identified, three (i.e., A114S, Y132H, and Y132F) have been reported previously (Morio et al., 2010). One strains (NCU_O080) only showed a synonymous mutation. Mutations in *ERG11* and the resultant amino acid changes are indicated in the **Table 5**. DNA sequencing of HS1 and HS2 of the drug target *FKS1* that is known to confer echinocandin resistance was performed on one *C. tropicalis* isolate (NCU_O081) and one *C*. *glabrata* isolate (NCU_B131). However, no mutation was detected in the hotspot regions of *FKS1* (**Supplementary Table 2**).

**Table 5:** Polymorphic sites in *ERG11* sequences.

Species	Strains number	Resistance to fungal drugs	Gene	Base mutation sits	Amino acid substitution
*C*. *albicans*	NCU_B138	Fluconazole	*ERG11*	C368T/T394C	T123I/Y132H
	NCU_B149	Voriconazole, Fluconazole	*ERG11*	G340T/T768C	A114S
	NCU_B154	Voriconazole, Fluconazole	*ERG11*	G340T/T768C	A114S
*C*. *parapsilosis*	NCU_O080	Voriconazole, Fluconazole	*ERG11*	T591C	None
	NCU_B136	Fluconazole	*ERG11*	A395T/T591C/G1193T	Y132F/R398I
*C*. *tropicalis*	NCU_B128	Voriconazole, Fluconazole	*ERG11*	T225C/G264A/A395T/C461T	Y132F/S154F
	NCU_B147	Voriconazole, Fluconazole	*ERG11*	G264A/C461T/G1362A/G1504A	S154F/D454N
*C*. *haemulonii*	NCU_B011	Voriconazole, Fluconazole	*ERG11*	A395T	Y132H

### Virulence Evaluation of the Invasive *Candida* spp. Isolates

Several virulence factors are involved in the pathogenesis of candidiasis, and they allow the fungal cells to escape or evade host defense mechanisms. These include phenotypic switching, biofilm formation, and secretion of multiple hydrolases (Neji et al., 2017). We investigated three virulence factors, namely, biofilm, caseinase, and hemolysin.

#### Caseinase Activity

**Figure 3:**
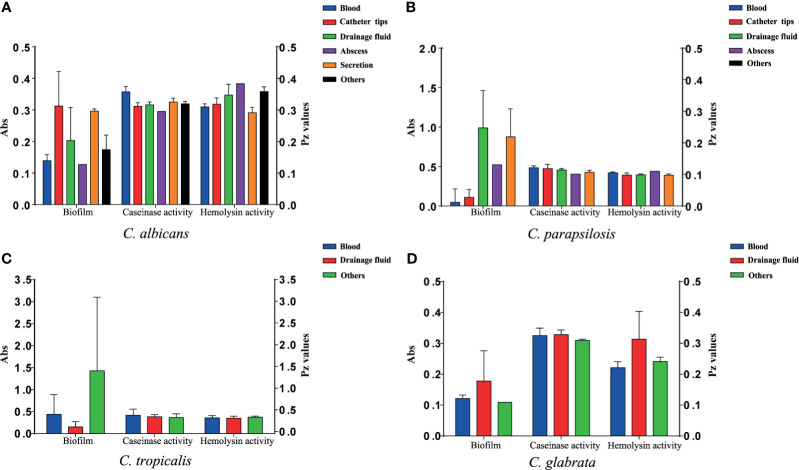
Virulence level (biofilm biomass, caseinase activity, and hemolysin activity) of the 107 *Candida* species isolates from different specimen sources. Y-axis (far left panel): biofilm biomass (positively correlated with virulence); Y-axis (far right panel): the Pz values of caseinase and hemolysin activities (negatively correlated with virulence). No significant differences were noted in these factors. **(A)** The virulence level of 40 *C. albicans* isolates from different specimen sources. **(B)** The virulence level of 37 *C. parapsilosis* isolates from different specimen sources. **(C)** The virulence level of 21 *C. tropicalis* isolates from different specimen sources. **(D)** The virulence level of 9 *C. glabrata* isolates from different specimen sources.

All 110 isolates were caseinase positive and 109 displayed strong activity. The mean Pz value in caseinase-positive *C*. *albicans* and *C*. *parapsilosis* isolates was 0.34 ± 0.06 and 0.47 ± 0.10, respectively, indicating a lower enzymatic activity in *C*. *parapsilosis* than that in *C*. *albicans* (**Table 2** and **Figure 2**). Of the 37 C. *parapsilosis* isolates, 36 displayed strong enzymatic activity, whereas one had moderate activity. All *C*. *tropicalis* (mean Pz = 0.40 ± 0.11) *and C*. *glabrata* isolates (mean Pz = 0.32 ± 0.02) were strong caseinase producers. No appreciable difference was noted in the caseinase activity from any of the specimen sources (**Figure 3**).

#### Hemolytic Activity

All of the tested isolates demonstrated strong hemolytic activity on human blood SDA plates with Pz values <0.69 (**Figure 2**). The mean Pz values for hemolytic activity were 0.32 ± 0.05, 0.41 ± 0.04, 0.36 ± 0.05, 0.25 ± 0.05, and 0.47 ± 0.10 for *C*. *albicans*, *C*. *parapsilosis*, *C*. *tropicalis*, and *C*. *glabrata*, respectively (**Table 2**). No noticeable differences were detected in the hemolytic activity of the strains from different sources (**Figure 3**).

#### Biofilm Formation

**Figure 4:**
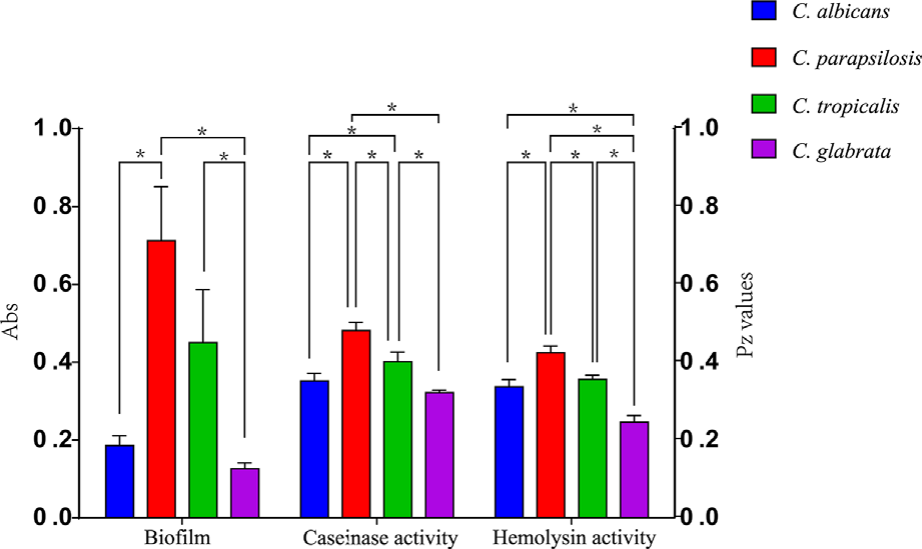
Comparison of the virulence levels (biofilm biomass, casein activity and hemolysin activity) of four *Candida* species. * indicates statistically significant (*p* < 0.05).

**Figure 2** and **Table 2** shows the results of biofilm quantification. Although *C*. *parapsilosis* isolates produced greater total biomass (average Abs_620_ = 0.712) than did *C*. *albicans* (average Abs_620_ = 0.186, *p* < 0.05) or *C*. *tropicalis* isolates (average Abs_620_ = 0.450; **Figure 4**), one *C*. *tropicalis* isolate (NCU_O081) yielded the greatest biomass (Abs_620_ = 2.611 ± 0.087), and this was significantly higher than that of *C*. *parapsilosis*. By contrast, all *C*. *glabrata* strains were low biofilm producers with Abs_620_ values ranging between 0.095 and 0.247. No significant differences were found in the extent of biofilm formation among all *C*. *glabrata* isolates (*p* > 0.05). Among *C*. *rugose* and *C*. *haemulonii*, it was impossible to derive any conclusions because of the small sample size. Furthermore, strains isolated from drainage fluid generally produced more biofilm than those isolated from blood, except for *C. tropicalis* species. *C*. *albicans* isolates obtained from catheter tips were the highest biofilm producers, whereas, for *C*. *parapsilosis*, these were the isolates obtained from drainage fluids (**Figure 3** and **Table 2**).

## Discussion

Invasive candidiasis is an emerging infection that is closely related to advances in medical technology, and it is widely recognized as a major cause of morbidity and mortality in high-risk groups such as immunosuppressed patients and those admitted to ICUs. At least 15 different species of *Candida* can cause human diseases, but a majority of the invasive infections are caused by five pathogens—*C*. *albicans*, *C*. *parapsilosis*, *C*. *glabrata*, *C*. *tropicalis*, and *C*. *krusei* (Pappas et al., 2018). Although *C*. *albicans* is currently the most common pathogen, non-*albicans Candida* spp. could collectively represent >50% of all bloodstream isolates in some regions (Pappas et al., 2018). A study of 141 clinical *Candida* specimens in Brazil reported that *C*. *albicans* is the most frequently isolated species (45.4%), followed by *C*. *parapsilosis* (28.4%), *C*. *tropicalis* (14.2%), and *C*. *glabrata* (1.4%) (Neufeld et al., 2015). A 6-year retrospective analysis of 351 patients with candidiasis showed that 48.1% of the candidemia episodes were due to *C*. *albicans*, followed by *C*. *parapsilosis* (25.1%) and *C*. *glabrata* (11.7%) (Ulu Kilic et al., 2017). In recent years, studies have described an increase in the incidence of *C*. *parapsilosis* infection (Ulu Kilic et al., 2017). We also show that *C*. *albicans* is the most prevalent disease-causing *Candida* spp., followed by *C*. *parapsilosis*, *C*. *tropicalis*, and *C*. *glabrata*, and that most isolates were obtained from patients hospitalized in ICUs.

RAPD analysis is a valuable tool for studying the genetic epidemiology of *Candida* infections, and multiple studies have confirmed its applicability and high discriminatory power for *Candida* genotyping at a local level (Paluchowska et al., 2014). Such accurate identification and molecular typing of *Candida* species provide valuable information for the prevention and control of nosocomial infections caused by these yeasts. Thus, the genetic diversity of 107 *Candida* species comprising *C*. *albicans* (n = 40), *C*. *parapsilosis* (n = 37), *C*. *tropicalis* (n = 21), and *C*. *glabrata* (n = 9), when an Sj value of 0.80 was defined as the limiting point, showed that *C*. *parapsilosis* and *C*. *tropicalis* isolates were more diverse than *C*. *albicans* isolates because of the greater range of Sj values in the dendrogram generated using the five primer pairs. The present study revealed two identical *C. albicans* isolates (NCU_B153 and NCU_B155) from the blood of two different patients admitted to ICU from different admission years. In addition, two *C. glabrata* isolates (NCU_B045 and NCU_B055) from different patients sharing the same ward showed similar genotype profiles. Notably, the aforementioned *C. glabrata* strains were isolated within the same month. These isolates may have come either from the patients, hospital personnel, medical devices, or environment, suggesting that *Candida* infection can spread exogenously.

Establishing antifungal susceptibility patterns among *Candida* species isolates from clinical specimens was an important aspect of our study. Fluconazole has remained the drug of choice for treating candidemia over several years now because of its efficacy against clinical *Candida* infection (Pappas et al., 2004; Autmizguine et al., 2018). However, the current treatment guidelines recommend the use of echinocandins as the first-line empirical treatment because of the increased resistance and treatment failure associated with the use of fluconazole (Bassetti et al., 2018; Zeng et al., 2019). Although we found that most isolates were sensitive to the echinocandins, one *C*. *glabrata* isolate was simultaneously resistant to three echinocandins and one *C*. *tropicalis* isolate was resistant to both anidulafungin and micafungin. Resistance to fluconazole (6.36%, 7/110) was high among the isolates tested here, and some strains displayed multiazole resistance, i.e., simultaneous resistance to both fluconazole and voriconazole among *C*. *albicans*, *C*. *parapsilosis*, and *C*. *tropicalis* isolates were 5% (2/40), 2.70% (1/37), and 9.52% (2/21), respectively. The appearance of multiazole-resistant strains could be associated with the high rates of azole usage in hospitals (36.2%, 38/105). The azole-resistant rates of patients with azole treatment to *C*. *albicans*, *C*. *parapsilosis*, *C*. *tropicalis* and *C*. *glabrata* were 30.0% (3/10), 25.0% (2/8), 25.0% (2/8), and 33.3% (1/3). Among these, 37.5% of the patients received long-course azole treatment for >15 days, which may be a risk factor for azole resistance. Although only two *C*. *rugose* strains and one *C. haemulonii* strain were isolated in our study, their high MICs against antifungal drugs could have been a cause for concern. In our study, the azole-resistance rate of *C. albicans* (7.5%, 3/10), *C. parapsilosis* (5.4%, 2/37), and *C. tropicalis* (9.5%, 2/21) were similar to those reported by Maubon et al. (Maubon et al., 2014). However, the resistance rate of *C. albicans* (7.5%*)* was significantly lower than that reported for a hospital in Southwest China (>20.0%) (Zeng et al., 2019). This discrepancy may be induced by the difference in the regional population, medical resources, and distribution of patient types.

DNA sequencing of the two drug target genes, *ERG11* and *FKS1*, was performed using drug-resistant strains. The pharmacological target of azoles is the enzyme 14-α-demethylase (encoded by *ERG11*), which is an enzyme for ergosterol biosynthesis (Spettel et al., 2019). The mutation leading to amino acid substitution in the *ERG11* sequence is one of the main mechanisms contributing to azole resistance in clinical isolates (Morio et al., 2010). As observed in previous studies, all *ERG11* mutations in the present study also occurred at three diffuse hotspot regions: amino acids 105–165, 266–287, and 405–488 (Marichal et al., 1999). The most common amino acid substitution in our study was 132aa (50%, 4/8) in the azole-resistant strains. A single Y132H substitution has been shown to reduce susceptibility to voriconazole and fluconazole, with MIC increased by four and eight times, respectively (Wang et al., 2005). The Y132H substitution was detected in one *C*. *albicans* isolate (NCU_B138) and one *C. haemulonii* isolate (NCU_B011). The Y132F substitution was also detected in a *C*. *parapsilosis* isolate (NCU_B136) and a *C*. *tropicalis* isolate (NCU_B128). A previous study confirmed that the Y132H substitution interfered with the interaction between the heme center of the enzyme and fluconazole, thereby reducing the affinity of the target enzyme to fluconazole, leading to fluconazole resistance (Kelly et al., 1999). Hence, our data support the involvement of Y132F and Y132H in azole resistance and their potential use as predictive markers of azole resistance. *FKS1* encode the β-1,3-glucan synthase, which is responsible for the synthesis of fungal cell wall and is the target enzyme of echinocandin (Sfeir et al., 2020). The most common cause of resistance is the serine-to-proline mutation in the HS1 region of *FKS1* at the position 654 (Sfeir et al., 2020). No mutation was detected in the HS1 and HS2 regions of *FKS1*, although some mutations in other regions of this gene may have been missed because of sequencing only the HS1 and HS2 regions. Notably, the finding of amino acid substitutions in these isolates does not indicate that there may not be other coexisting mechanisms of antifungal resistance, such as the overexpression of *ERG11*, *CDR1*, and/or *MDR1*, although these points were not proved in our study.

Several virulence factors in *Candida* species are indispensable for their ability to cause disease, and these include extracellular hydrolytic enzymes, particularly proteases, phospholipases, and hemolysins, which facilitate food acquisition, adherence, colonization, invasion, diffusion, and escape from host immune responses (Abbes et al., 2017; Figueiredo-Carvalho et al., 2017). Accurate identification of putative virulence factors in *Candida* spp. is critical for predicting the response of antifungal drugs and detecting the emergence of strains with greater resistance. França et al. have reported that although 68% of patients with candidiasis had been prescribed antifungal therapy with a favorable susceptibility profile, the mortality rate was 56%, implying that other factors are involved in determining patient prognosis (França et al., 2008). Two such factors may be biofilm formation and the expression of virulence-associated factors. Biofilm formation is a major virulence factor (Mba and Nweze, 2020) because, when generated on host tissues and indwelling medical devices such as central venous catheters, they hinder drug diffusion and render infection eradication extremely difficult (Chen et al., 2020). We compared certain virulence factors among *Candida* species that are detectable in a laboratory environment, such as caseinase, hemolysin, and biofilm production, to assess the pathogenic potential of *Candida* species.

Proteases can degrade the host epithelial and mucosal barrier proteins such as albumin, collagen, and mucin. They also help *Candida* resist attacks by host immune system thorough the degradation of antibodies, complements, and cytokines (Borst and Fluit, 2003; Neji et al., 2017). Casein has also been widely reported as a substrate to evaluate the protease activity of pathogenic strains (Fernandes et al., 2012; Neji et al., 2017). Our results show that all the *Candida* spp. were caseinase producers with a strong enzymatic activity (99.09%). Furthermore, all *C*. *glabrata* strains showed a strong enzymatic activity and our results are consistent with findings reported by Abbes et al. who also tested *C*. *glabrata* isolates (Abbes et al., 2017). However, these results are in contrast to those described by Figueiredo-Carvalho et al. who reported no caseinase activity in 91 C. *glabrata* isolates (Figueiredo-Carvalho et al., 2017). Using casein as a substrate, all isolates of *C*. *albicans*, *C*. *parapsilosis*, *C*. *tropicalis*, *C*. *haemulonii*, and *C*. *rugose* were found to be proteinase positive. Lower caseinase activities were detected for *C. parapsilosis* than for *C*. *albicans*, *C*. *tropicalis*, and C. *glabrata* isolates with a statistically significant difference (**Figure 4**); these differences were independent of the specimen sources and genotypes (**Figure 4**; **Supplementary Figure 1**).

Hemolytic activity is another virulence factor exhibited by pathogenic microorganisms, which enables fungal pathogens to utilize hemoglobin as an iron source for growth in the iron-scarce host environment (Weissman et al., 2021). Previous studies have shown a close relationship between cellular iron and drug susceptibility in *C*. *albicans* (Prasad et al., 2006), and iron uptake mechanisms have also been reported to be necessary for the virulence of C. *glabrata* (Figueiredo-Carvalho et al., 2017). In our study, all *Candida* spp. showed strong hemolytic activity. Moreover, *C*. *tropicalis* exhibited greater hemolysin production than *C*. *glabrata*. We noted that the hemolytic activity of *C*. *albicans* isolates was higher than that of *C*. *parapsilosis* isolates; these findings are inconsistent with those of previous studies (**Figure 4**) (Chin et al., 2013; Neji et al., 2017).

Several studies have established the importance of biofilm formation in clinical infections due to *Candida* strains (Nett and Andes, 2006; Akers et al., 2015). Estivill et al. found that biofilm formation was observed on implantable medical devices and all *Candida* strains tested (including *C*. *albicans*, *C*. *parapsilosis*, *C*. *tropicalis*, *C*. *glabrata*, and *C*. *krusei*) were able to form biofilms (Estivill et al., 2011). Our results correspond with those reported previously, especially that *C*. *glabrata* showed no biofilm production (Marak and Dhanashree, 2018). This is likely, in part, to be related to its intrinsic inability to form hyphae (Seidler et al., 2006). More *C*. *albicans* isolates produced biofilms than *C*. *parapsilosis* or *C*. *tropicalis* isolates, but *C*. *rugose* and *C*. *haemulonii* showed no biofilm production in our study. We also performed subgroup analyses by specimen source and found that isolates from drainage fluids exhibited greater biofilm formation than those originating from blood, except among *C*. *tropicalis* isolates.

The association between clusters and virulence factors was compared (**Supplementary Figure 1**). No significant difference was noted in the hemolytic and caseinase activities among the strains of different genotypes, although their biofilm production was found to vary with the different clusters. For instance, for *C. parapsilosis*, three clusters (K–M) showed higher biofilm formation ability than the others (**Supplementary Figure 1B**). A similar situation was also noted for *C*. *tropicalis*, although the difference failed to reach a statistical significance (**Supplementary Figure 1C**). We also compared the virulence factors of the isolates from different specimen sources, although no obvious correlation was recorded. Interestingly, a correlation was noted between the RAPD genotypes and antifungal resistance. Moreover, 75% of the *C. albicans* from cluster F demonstrated azole resistance, whereas only two azole-resistant *C. tropicalis* strains belonged to the cluster Y (**Figure 2A**). Moreover, 66.7% of the *C. tropicalis* strains, which were S-DD to azole drugs, were allocated into cluster W (**Figure 2C**). These findings together suggest that RAPD results allow the selection of drug-resistant isolates with diverse genetic backgrounds, which has significance in clinical medication and applications.

## Conclusion

To summarize, this study is able to investigate the molecular relationship and genetic diversity among 110 clinical *Candida* isolates obtained from hospital inpatients admitted in the Nanchang region of China. Although these data are expected to help understand the epidemiology of *Candida* species in Nanchang City, our results also contribute toward a greater understanding of the pathogenicity of *Candida* species in patients with candidiasis and the susceptibility of *Candida* strains to the most commonly used antifungal drugs; the latter may also help prevent the occurrence and outbreak of *Candida* infections. Furthermore, our data provide experimental evidence for designing appropriate clinical monitoring and treatment strategies for candidiasis. Because of the rising incidence of *Candida* infections and their resistance to antifungal drugs, further studies are required to develop treatment strategies against *Candida* strains.

## Data Availability Statement

The sequencing results of ERG11 and FKS1 in the study are deposited in the NCBI BankIt database under accession numbers MZ711431 to MZ711438 and MZ711439 to MZ711442, respectively.

## Ethics Statement

The ethics committee of the First Affiliated Hospital of Nanchang University approved this study (approval no. 2014036).

## Author Contributions

LZ, NH, QL, XY, and XH designed the study. JC, NH, HX, YZ,YH, and JT performed the experiments and analyses, and wrote the original draft. LZ and XH edited the manuscript. All authors contributed to the article and approved the submitted version.

## Funding

This work was supported by the National Natural Science Foundation of China (32060040 and 31760261), the Natural Science Foundation of Jiangxi Province (20202BABL216084, 20192ACBL21042, 20202BAB216045, 20192BBG70067, and 20204BCJL23054), the Science and Technology Research Project of Education Department of Jiangxi Province (GJJ180130), and the Major Science and Technology Project of Jiangxi Province (20181BBG70030).

## Conflict of Interest

The authors declare that the research was conducted in the absence of any commercial or financial relationships that could be construed as a potential conflict of interest.

## Publisher’s Note

All claims expressed in this article are solely those of the authors and do not necessarily represent those of their affiliated organizations, or those of the publisher, the editors and the reviewers. Any product that may be evaluated in this article, or claim that may be made by its manufacturer, is not guaranteed or endorsed by the publisher.

## Supplementary Material

Supplementary Figure 1 Comparison of the virulence levels (biofilm biomass, casein activity, and hemolysin activity) of three *Candida* species isolates by genotypic relatedness. Y-axis (far left panel): biofilm biomass (positively correlated with virulence); Y-axis (far right panel): the Pz values of caseinase and hemolysin activities (negatively correlated with virulence). No significant differences can be noted in this figure. **(A)** The virulence level of 40 C*. albicans* isolates from different gene clusters; **(B)** The virulence level of 37 C*. parapsilosis* isolates from different gene clusters; **(C)** The virulence level of 21 C*. tropicalis* isolates from different gene clusters.
