# Supplementary figures and images for: Molecular Epidemiology, Antifungal Susceptibility, and Virulence Evaluation of Candida Isolates Causing Invasive Infection in a Tertiary Care Teaching Hospital
Source: Front Cell Infect Microbiol. 2021 Sep 15;11:721439. doi: 10.3389/fcimb.2021.721439 (PMC8479822; doi:10.3389/fcimb.2021.721439)

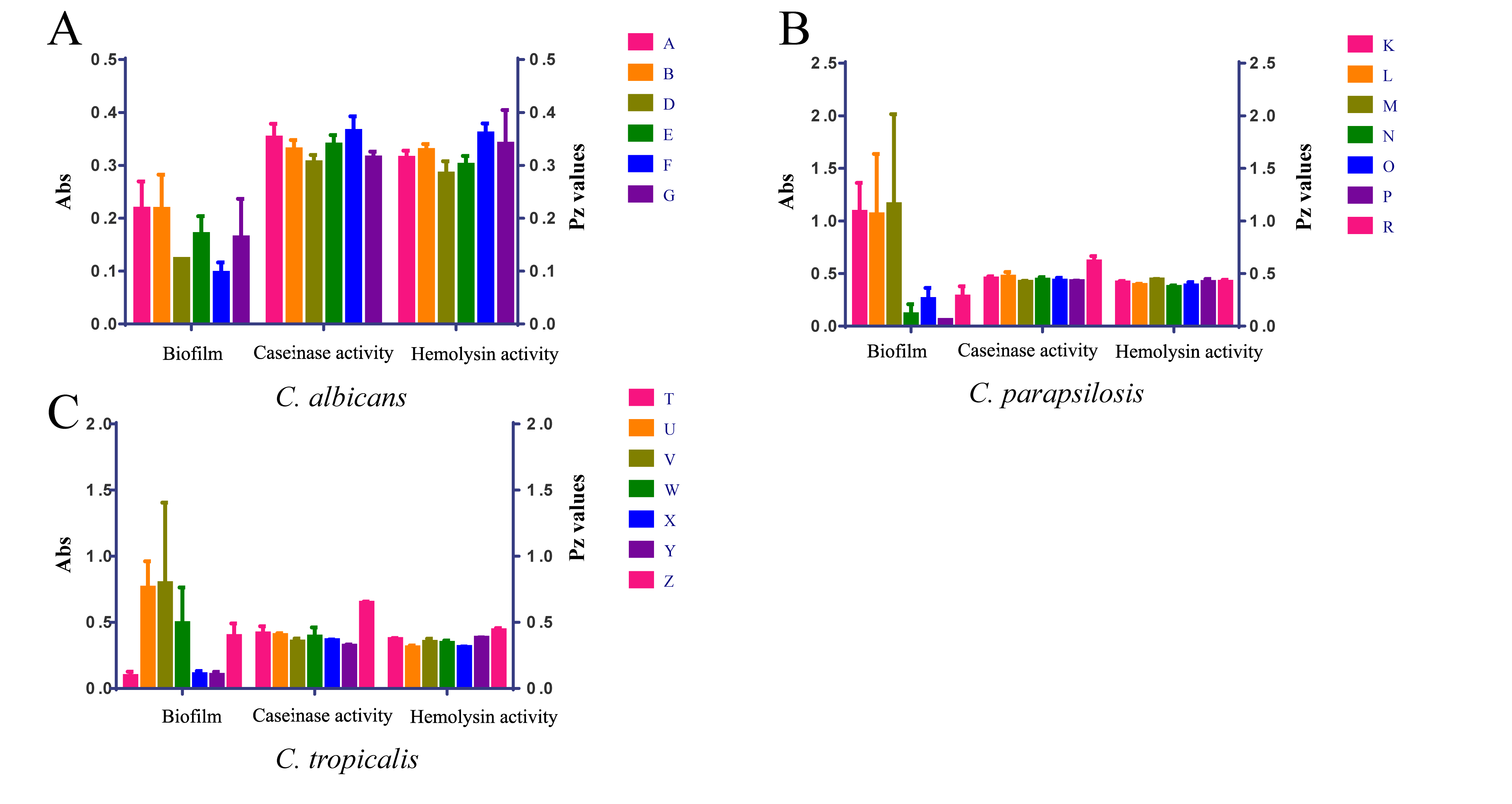

Supplement: Supplementary Figure 1 — Comparison of the virulence levels (biofilm biomass, casein activity, and hemolysin activity) of three Candida species isolates by genotypic relatedness. Y-axis (far left panel): biofilm biomass (positively correlated with virulence); Y-axis (far right panel): the Pz values of caseinase and hemolysin activities (negatively correlated with virulence). No significant differences can be noted in this figure. (A) The virulence level of 40 C. albicans isolates from different gene clusters; (B) The virulence level of 37 C. parapsilosis isolates from different gene clusters; (C) The virulence level of 21 C. tropicalis isolates from different gene clusters. [file Image_1.tif]
