# Supplementary material for: Molecular Epidemiology, Antifungal Susceptibility, and Virulence Evaluation of Candida Isolates Causing Invasive Infection in a Tertiary Care Teaching Hospital
Source: Front Cell Infect Microbiol. 2021 Sep 15;11:721439. doi: 10.3389/fcimb.2021.721439 (PMC8479822; doi:10.3389/fcimb.2021.721439)
Supplement: Supplementary file 3 [file Table_1.docx]

**Supplementary Table 1:** **List of primers used for *ERG11* and *FKS1* PCR amplification and sequencing.**

| **Organism** | **Primer name** | **Sequence (5'-3')** | **Product length (bp)** | **Purpose** | **Gene ID** |
| --- | --- | --- | --- | --- | --- |
| *C. albicans* | *Ca-ERG11*-F1 | GAAAGGGAATTCAATCGTTATTC | 1018 | Ca*ERG11* sequencing | 3641571 |
|  | *Ca-ERG11*-R1 | GTTGACCACCCATAAGAATACC |  |  |  |
|  | *Ca-ERG11*-F2 | CATTATTGGAGACGTGATGCTGC | 1095 |  |  |
|  | *Ca-ERG11*-R2 | CCAGTGGACAAAAACCATCAAC |  |  |  |
| *C. parapsilosisr* | *Cp-ERG11*-F1 | CATCCAAAGACCTTAGCTGAG | 1159 | Cp*ERG11* sequencing | GQ302972.1 |
|  | *Cp-ERG11*-R1 | CATCCAAGTTGCCACCTTTAC |  |  |  |
|  | *Cp-ERG11*-F2 | GGTGTTAAAATGACTGATCAGG | 945 |  |  |
|  | *Cp-ERG11*-R2 | CAGAAGCGACGTAGAATTGTG |  |  |  |
| *C. tropicalis* | *CT-ERG11*-F1 | TCACAGTTATAGACCCACAAGG | 878 | Ct*ERG11* sequencing | M23673 |
|  | *CT-ERG11*-R1 | TCACCGCTTTCTCTTCTTCTCT |  |  |  |
|  | *CT-ERG11*-F2 | CAAAACCAAGGAAAGAGACCATG | 1583 |  |  |
|  | *CT-ERG11*-R2 | TCCCAAGACATCAAACCCTG |  |  |  |
|  | *CT-FKS1*-F1 | GTATTCCAAGTCTGCTTACGCTG | 571 | Ct*FKS1* HS1 sequencing | EU676168 |
|  | *CT-FKS1*-R1 | CCTTTGGTTTGTACTTGATTTCC |  |  |  |
|  | *CT-FKS1*-F2 | CACTACCAAGATTGGTGCTGG | 701 | Ct*FKS1* HS2 sequencing |  |
|  | *CT-FKS1*-R2 | GTGGGTTGAAAATAAATGGTGAG |  |  |  |
| *C. glabrata* | *Cg-FKS1*-F1 | CCATTGGGTGGTCTGTTCACG | 770 | Cg*FKS1* HS1 sequencing | XM_446406 |
|  | *Cg-FKS1*-R1 | GGCATGTTGTCAACTGGCAATG |  |  |  |
|  | *Cg-FKS1*-F2 | GGTATTTCAAAGGCTCAAAAGGG | 835 | Cg*FKS1* HS2 sequencing |  |
|  | *Cg-FKS1*-R2 | ATGGAGAGAACAGCAGGGCG |  |  |  |
| *C. haemulonis* | *Ch-ERG11*-F1 | CAATTGCACATTCGCTGAAC | 1085 | Ch*ERG11* sequencing | XM_025486744.1 |
|  | *Ch-ERG11*-R1 | CATCTTAACGCCATCTTTATAGG |  |  |  |
|  | *Ch-ERG11*-F2 | CCGTGAACTTTGTATTCCCAC | 1037 |  |  |
|  | *Ch-ERG11*-R2 | CATGGTTGTGATGTGACTTCTC |  |  |  |
