# Supplementary material for: Molecular Epidemiology, Antifungal Susceptibility, and Virulence Evaluation of Candida Isolates Causing Invasive Infection in a Tertiary Care Teaching Hospital
Source: Front Cell Infect Microbiol. 2021 Sep 15;11:721439. doi: 10.3389/fcimb.2021.721439 (PMC8479822; doi:10.3389/fcimb.2021.721439)
Supplement: Supplementary file 4 [file Table_2.docx]

**Supplementary Table 2: Gene sequencing of HS1 and HS2 region of *FKS1* gene.**

| **Species** | **Strains** | **Region** | **DNA Sequence** | **Protein** | **Amino acid substitution** | **References** |
| --- | --- | --- | --- | --- | --- | --- |
| *C*. *tropicalis* | ATCC 750 Ref strain | HS1 | TTCTTGACTTTGTCTTTAAGAGATCCA | FLTLSLRDP | - | (Desnos-Ollivier et al., 2008) |
|  |  | HS2 | AATCTTTCTCCAGCTGTTGATTGG | NLSPAVDW |  |  |
|  | NCU_O081 | HS1 | TTCTTGACTTTGTCTTTAAGAGATCCA | FLTLSLRDP | None |  |
|  |  | HS2 | AATCTTTCTCCAGCTGTTGATTGG | NLSPAVDW |  |  |
| *C*. *glabrata* | CBS138  Ref strain | HS1 | TTCTTGATT CTATCTCTAAGAGATCCA | FLILSLRDP | - | (Vatanshenassan et al., 2019) |
|  |  | HS2 | GACTGGGTCAGACGTTACACATTA | DWVRRYTL |  |  |
|  | NCU_B131 | HS1 | TTCTTGATTCTATCTCTAAGAGATCCA | FLILSLRDP | None |  |
|  |  | HS2 | GACTGGGTCAGACGTTACACATTA | DWVRRYTL |  |  |

**Reference:**

Desnos-Ollivier, M., Bretagne, S., Raoux, D., Hoinard, D., Dromer, F., and Dannaoui, E. (2008). Mutations in the fks1 gene in Candida albicans, C. tropicalis, and C. krusei correlate with elevated caspofungin MICs uncovered in AM3 medium using the method of the European Committee on Antibiotic Susceptibility Testing. *Antimicrobial agents and chemotherapy* 52, 3092-3098. doi: 10.1128/AAC.00088-08

Vatanshenassan, M., Arastehfar, A., Boekhout, T., Berman, J., Lass-Flörl, C., Sparbier, K., et al. (2019). Anidulafungin Susceptibility Testing of Candida glabrata Isolates from Blood Cultures by the MALDI Biotyper Antibiotic (Antifungal) Susceptibility Test Rapid Assay. *Antimicrobial agents and chemotherapy* 63. doi: 10.1128/AAC.00554-19
